# Retraction: Inhibition of microRNA-299-5p sensitizes glioblastoma cells to temozolomide via the MAPK/ERK signaling pathway

**DOI:** 10.1042/BSR-2018-1051_RET

**Published:** 2024-01-25

**Keywords:** Glioblastoma (GBM), Golgi phosphoprotein 3 (GOLPH3), invasion, miR-299-5p, Proliferation, temozolomide

This article is being retracted from *Bioscience Reports* at the request of the Editor-in-Chief and the Editorial Board following a request from the authors to correct the Western blots in Figures 5, which at the time of publication contained duplications. Readers also identified that the images in Figure 2A also appear in Zeng et al. 2018 (doi: 10.1038/s41419-018-0343-1).

The authors were asked to provide the raw images for the Western blots as part of the correction process and, upon receipt, the Editorial Office identified signs of digital editing and thus no longer has confidence in the validity of these results. The authors were contacted regarding the concerns and provided another set of raw Western blot images, however these also showed signs of digital editing. When the authors were alerted to this in the second set of images, they stated that the Western blot experiments were conducted by a co-author who has since resigned, and that the original Western blots are missing. The authors were asked to agree to the retraction but did not respond.

